# Endocan Levels in Peripheral Blood Predict Outcomes of Acute Respiratory Distress Syndrome

**DOI:** 10.1155/2014/625180

**Published:** 2014-07-16

**Authors:** Ling Tang, Ying Zhao, Daoxin Wang, Wang Deng, Changyi Li, Qi Li, Shicong Huang, Chang Shu

**Affiliations:** ^1^Department of Pulmonary and Critical Care Medicine, Second Affiliated Hospital of Chongqing Medical University, Chongqing 400010, China; ^2^Respiratory Department, Xinqiao Hospital, Chongqing 400037, China; ^3^Respiratory Department, First Affiliated Hospital of Chongqing Medical University, Chongqing 400042, China; ^4^Respiratory Department, Tong Liang County People's Hospital, Chongqing 402560, China

## Abstract

*Purpose.* To investigate the prognostic significance of endocan, compared with procalcitonin (PCT), C-reactive protein (CRP),white blood cells (WBC), neutrophils (N), and clinical severity scores in patients with ARDS.* Methods.* A total of 42 patients with ARDS were initially enrolled, and there were 20 nonsurvivors and 22 survivors based on hospital mortality. Plasma levels of biomarkers were measured and the acute physiology and chronic health evaluation II (APACHE II) was calculated on day 1 after the patient met the defining criteria of ARDS.* Results.* Endocan levels significantly correlated with the APACHE II score in the ARDS group (*r* = 0.676, *P* = 0.000, *n* = 42). Of 42 individuals with ARDS, 20 were dead, and endocan was significantly higher in nonsurvivors than in survivors (median (IQR) 5.01 (2.98–8.44) versus 3.01 (2.36–4.36) ng/mL, *P* = 0.017). According to the results of the ROC-curve analysis and COX proportional hazards models, endocan can predict mortality of ARDS independently with a hazard ratio of 1.374 (95% CI, 1.150–1.641) and an area of receiver operator characteristic curve (AUROC) of 0.715 (*P* = 0.017). Moreover, endocan can predict the multiple-organ dysfunction of ARDS.* Conclusion.* Endocan is a promising biomarker to predict the disease severity and mortality in patients with ARDS.

## 1. Introduction

Acute respiratory distress syndrome (ARDS) is characterized by alveolar epithelial and vascular endothelial injury in the lungs that is triggered by a wide range of predisposing conditions such as pneumonia, sepsis, and trauma [1–23]. It is a frequent cause of ICU admission and has a high rate of mortality and morbidity. As it remains challenging to identify patients who are at the highest risk of developing these syndromes and to differentiate these syndromes from other causes of acute respiratory failure, many studies have focused on biomarkers to identify patients with ARDS to predict those who are unlikely to have a positive outcome and create evidence-based therapies. Until now, four categories of biomarkers have been studied including inflammatory cytokines (IL-6, IL-8) [4, 5], coagulation proteins (PAI-1, protein C) [6, 7], epithelial proteins (KL-6, SP-D, RAGE) [8–10], and endothelial proteins (Ang-2, ICAM-1, vWF) [11–13]. Despite recent advances in our understanding of biomarkers associated with either diagnosis of ARDS in the at-risk population or ARDS-related mortality, researchers continue to explore a reliable ARDS biomarker.

Endocan, also called endothelial cell-specific molecule-1, is a soluble 50 kDa dermatan sulfate proteoglycan that is secreted from pulmonary and kidney vascular endothelial cells [14]. Endocan is stable at low levels in the blood of healthy subjects and can be measured in serum [15]. In vitro, endocan can bind directly to the integrin CD11a-CD18 (LFA-1) and block binding to the intercellular adhesion molecule-1 (ICAM-1) [16], consequently inhibit leukocyte-endothelial cell adhesion, and reduce the excessive leukocyte recruitment into the lungs. Some studies showed that endocan can be acknowledged as a good marker of endothelial dysfunction and multiple-organ dysfunction in sepsis, and it can be accepted as a good marker of survival prognosis in sepsis [17, 18]. However, few study investigated the performance of endocan in ARDS. Therefore, the primary aim of our study was to test whether endocan is useful for the prognosis of ARDS.

## 2. Material and Methods

### 2.1. Participants

This was a multicenter clinical study conducted at the Second Affiliated Hospital of Chongqing Medical University, First Affiliated Hospital of Chongqing Medical University and Xinqiao Hospital from January 2012 to August 2013. We enrolled 42 critical ill adult patients with acute respiratory distress syndrome [19], which was triggered by some predisposing conditions such as pulmonary infection, sepsis, aspiration, and blood transfusion. Patients were followed until death in hospital or discharge home and were then defined as nonsurvivors or survivors. Pulmonary infection is the main cause of ARDS in china. Diffuse alveolar damage is the pathological process of ARDS with pulmonary infection, which differs from pneumonia without ARDS. To analyse the difference between pneumonia patients with ARDS and those without ARDS, we recruited 44 pneumonia patients without ARDS, compared with 35 pneumonia patients with ARDS.

Patients were excluded if they were less than 18 years old or pregnant or if they had a coexisting malignancy. The study protocol had been reviewed and approved by the local Institutional Review Board, and written informed consent was obtained from either the patient or from each patient's next of kin or legal representative before enrollment.

### 2.2. Sample Collection

Plasma specimens were obtained from patients with ARDS as soon as possible after the patient met defining criteria, but those obtained more than 24 hours after admission were excluded.

### 2.3. Data Collection

Demographic characteristics and clinical data including age, gender, etiology of ARDS, and admission comorbidities were recorded from each subject. The acute physiology and chronic health evaluation (APACHE) II score [20] and PaO^2^/FiO^2^ were recorded based on the lowest value at onset. We also recorded the duration of mechanical ventilation and length of stay in the Intensive Care Unit and length of hospital stay.

### 2.4. Measurements

When patients admitted, the WBCs, neutrophils, PCT, and CRP concentrations were routinely inspected and we recorded the results. We used a sandwich-based enzyme-linked immunosorbent assay (ELISA; LUNGINNOV Systems, Lille, France) to measure endocan plasma concentrations in duplicate.

### 2.5. Statistical Analysis

For continuous variables, descriptive results were presented as the median (IQR) unless stated otherwise; we used the Student* t*-test for data that followed normal distribution and the Mann-Whitney* U*-test for those that were not normally distributed. Categorical variables were compared using the Fisher exact test. Receiver operating characteristic (ROC) curves were computed and areas under the curves were used to evaluate the predictive value of biomarkers for ARDS and the ability of the model to distinguish the survivor group from the nonsurvivor group. To assess the relationship between two variables, Spearman rank analysis was used for variables that followed abnormal distribution and Pearson correlation analysis was used for those with normal distribution. To identify risk factors for hospital mortality, we used a multivariate Cox proportional hazards regression model with forward stepwise selection procedures. A *P*-value less than 0.05 in the univariate analysis was required for a variable to enter the multivariate model. All analyses were performed using SPSS 19.0 for Microsoft Windows (SPSS Inc., Chicago, IL). MedCalcR version 4.20.011 (Frank Schoonjans, Mariakerke, Belgium) was used to compare the ROC curves and GraphPad 5.0 software was used to draw a correlation analysis figure. A *P*-value of less than 0.05 was considered statistically significant.

## 3. Results

### 3.1. Baseline Characteristics of the Patients with ARDS

42 critical ill adult patients with ARDS were initially enrolled in this study.  Table 1 summarizes the basic demographics. Defined by the survival status after patients with ARDS discharge home, there were 22 survivors and 20 nonsurvivors in this cohort. The main etiology of ARDS was pulmonary infection, included bacterial pneumonia, virus pneumonia, and active pulmonary tuberculosis, while the other causes included aspiration, blood transfusion, and extra pulmonary infection. Comparing survivors with nonsurvivors, we found that nonsurvivors were slightly older than survivors (*P* = 0.064) and nonsurvivors had a higher APACHE II score (median 24 versus 21; *P* = 0.03) and lower PaO^2^/FiO^2^ ratios than survivors (median 89.5 versus 131; *P* < 0.001). Furthermore, catastrophic complications including shock, acute renal failure, hepatic dysfunction, and coagulopathy were more common among nonsurvivors.

### 3.2. Plasma Biomarker Levels in Patients with ARDS

All patients were classified in 3 Berlin's subclasses: mild, moderate, or severe ARDS, but mild group had no patient in our study. Endocan levels had no significant statistical significance between moderate ARDS group (median (IQR) 3.21 (2.38–4.96) ng/mL; *n* = 23) and severe group (median (IQR)4.35(2.97–7.28) ng/mL; *n* = 19) (*P* = 0.176).

In the ARDS group, the median endocan levels in nonsurvivor plasma were significantly higher than in survivor plasma (median (IQR) 5.01 (2.98–8.44) ng/mL versus 3.01 (2.36–4.36), respectively; *P* = 0.017). Meanwhile, procalcitonin (PCT) also showed significant statistical significance when comparing the nonsurvivors group with the survivors group (median (IQR) 9.26 (3.88–16.46) ng/mL versus 2.59 (0.95–6.25) ng/mL, respectively; *P* = 0.007). However, WBCs, neutrophil counts, and CRP were unable to distinguish survivors from nonsurvivors, as shown in Table 2.

### 3.3. The Correlation between Circulation Endocan Levels and the Other Biomarkers in ARDS Group

**Figure 1:**
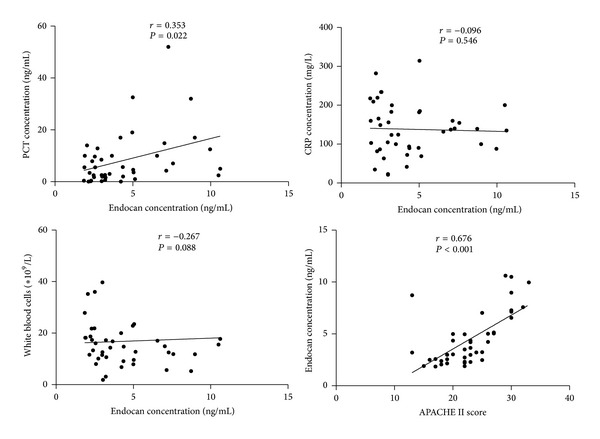
Correlations of plasma endocan with procalcitonin (PCT), C-reactive protein (CRP), white blood cells (WBC), and APACHE II in 42 patients with acute respiratory distress syndrome (Spearman rank analysis). *r* represents Spearman's correlation coefficients, and *P* value less than 0.05 was considered statistically significant.

Figure 1 showed the correlation between endocan expression levels and the other biomarkers of ARDS subjects. There were significant correlations between endocan levels and APACHE II (*r* = 0.676, *P* < 0.001, *n* = 42), and PCT (*r* = 0.353, *P* = 0.02, *n* = 42). However, correlations between endocan levels and CRP (*r* = −0.096, *P* = 0.546, *n* = 42), white blood cells (*r* = −0.267, *P* = 0.088, *n* = 42), and neutrophil counts (*r* = −0.278, *P* = 0.075, *n* = 42) were not statistically significant. Additionally, we also found that endocan levels as well as PCT, CRP, and WBC counts did not exhibit a significant correlation with the duration of mechanical ventilation and the length of hospital stay (results were not shown).

### 3.4. Endocan Levels Predict the Mortality of Patients with ARDS

**Figure 2:**
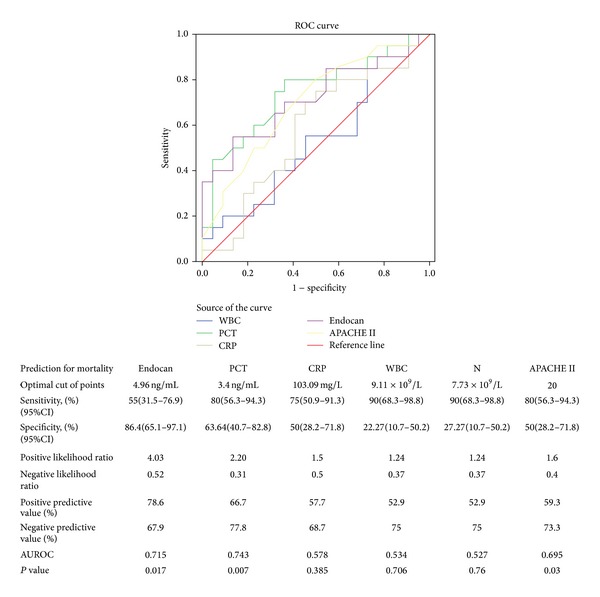
Motality prediction by plasma levels of endocan, PCT, CRP, WBC and Neutrophil counts and APACHE II scores in patients with acute respiratory distress syndrome using the receiver operating characteristic (ROC) curves.The optimal cutoff points for each plasma biomarker level and severity score were listed in the attached table, *P* value less than 0.05 was considered statistically significant.

To investigate the predictive properties of endocan levels regarding survival, we use the ROC-curve analysis and forward stepwise multivariate Cox regression. As shown in Figure 2, the area under the ROC curve (AUROC) of endocan was 0.715 (95% CI, 0.555–0.875); this value was similar to the PCT (0.743, 95% CI, 0.59–0.896) and slightly higher than the APACHE II score (0.695, 95% CI, 0.535–0.856). Patients with endocan levels above 4.96 ng/mL (the optimal cutoff points) had a particularly negative outcome with a sensitivity of 55% and a specificity of 86.4%. Further, the optimal cutoff points of other parameters for predicting the mortality are 3.4 ng/mL for PCT, 103.09 mg/L for CRP, 9.11 × 10^9^/L for WBC, 7.73 × 10^9^/L for neutrophil counts, and 20 for the APACHE II score. Furthermore, the positive predictive value, negative predictive value, and positive likelihood ratio and negative likelihood ratio for each parameter by individual threshold are listed below the graph in Figure 2.

For further risk assessment, we performed a forward stepwise multivariate Cox regression to compute the univariate analysis and hazard ratios, which are displayed in Table 3. Among these parameters, only endocan and PaO^2^/FiO^2^ were independently associated with mortality. The hazard ratio for endocan was 1.374 (95% CI, 1.150–1.641) and 0.958 (95% CI, 0.938–0.978) for PaO^2^/FiO^2^.

### 3.5. Endocan Levels Predict Multiple-Organ Dysfunction in Patients with ARDS

We also compared plasma markers using the area under the ROC curves to predict multiple-organ dysfunction (Table 4). The results showed that baseline endocan levels are able to predict shock, renal failure, and coagulopathy with high values. According to the optimal cutoff point determined from the ROC curve, patients were stratified into a high plasma endocan level (≥4.96 ng/mL) group and a low endocan level (<4.96 ng/mL) group. We found that endocan can predict the incidence of shock and renal failure with high levels, but it is unable to predict the occurrence of hepatic dysfunction and coagulopathy, which was the same within a 1-week period (Table 5).

### 3.6. Difference Analysis between Pulmonary Infection with ARDS and without ARDS

Demographic and baseline clinical characteristics of them were shown in Table 6. Endocan was statistically significantly higher among pneumonia patients who developed ARDS than those who did not (median (IQR) 3.22 (2.47–5.14) ng/mL versus 2.45 (2.23–2.79) ng/mL, resp.; *P* = 0.000). But PCT, CRP, WBCs, and neutrophil counts were unable to distinguish them. Meanwhile, endocan can differentiate the above two groups with a sensitivity of 65.71%, a specificity of 86.36%, and an area under receiver operator characteristic curve (AUROC) of 0.735, which was superior to the other biomarkers (Table 7).

## 4. Discussion

Pulmonary infection is the primary risk factor of ARDS in china, but not every patient with pneumonia would develop into acute lung injury, so which person is at risk for ARDS is unknown. In our observational cohort study, levels of plasma endocan were significantly elevated in pneumonia patients with ARDS compared with those without ARDS. As we all know, neutrophils play a critical role in the pathogenesis of ARDS and when activated they release harmful mediators including cytokines, proteases, reactive oxygen species, and matrix metalloproteinases leading to further damage. However, endocan was shown to inhibit the interaction between intercellular adhesion molecule-1 (ICAM-1) and the integrin (lymphocyte function-associated antigen-1) LFA-1 on leukocytes,and can modulate LFA-1 mediated leukocyte functions, such as the firm adhesion of leukocytes to the endothelium and the leukocyte transmigration [16]. In vitro, bacteria endotoxin LPS and proinflammatory cytokines such as IL-1*β* and TNF-*α* induce the synthesis and the release of endocan by HUVECs [14, 15] and sustained release of endocan. The sustained hypersecretion of endocan stimulated by TNF-*α* and LPS may be consistent with the high level of serum endocan in patients with fatal outcome, observed in our patients with ARDS and also in septic patients [17, 18]. However, the presence of endocan in a storage form within the endothelial cells [15] may also suggest that endocan release could be partly due to endothelial cell injury. Therefore, endocan may represent a novel endothelial cell dysfunction marker.

In the last decades, the focus of biomarker research in ARDS got significant progress. Ms. Terpstra and Dr. Aman conducted a systematic review and meta-analysis of all studies on plasma biomarkers associated with either diagnosis of ARDS in the at-risk population or ARDS-related mortality.They showed that increased plasma levels of KL-6, LDH, sRAGE, and vWF are most strongly associated with ARDS diagnosis in the at-risk population, whereas the strongest association with ARDS mortality was found for IL-4, IL-2, Ang-2, and KL-6 [21]. However, we need continue to explore a reliable biomarker of ARDS to enrich our knowledge. In our study, patients with endocan levels above 4.96 ng/mL had a poor chance for survival and were more likely to develop septic shock and renal failure. Meanwhile, endocan can predict mortality of ARDS independently with a hazard ratio of 1.374, specificity of 86.4%, and an AUROC of 0.715. Although its sensitivity was low, it does not exclude high performance of endocan when measured in other compartments, such as bronchoalveolar fluid or exhaled gas, or combining endocan with the other biomarkers of ARDS to improve the accuracy of the prognosis of ARDS. PCT is a marker to improve the diagnosis of bacterial infections and to guide antibiotic therapy [22]. In our study, PCT had higher sensitivity and similar AUROC but lower specificity to predict the mortality of ARDS, which may be related to most participants with severe lung infections in our study. However, PCT had no discriminative power for prediction between pulmonary infection with ARDS and without ARDS.

Multiple-organ dysfunction (MODS) indicates the exacerbation of patients with ARDS.

Endocan levels above 4.96 ng/mL were more likely to develop into septic shock and renal failure, in which the AUROC of endocan was 0.772 for septic shock and 0.714 for renal failure. By contrast, PCT, CRP, and WBC did not show discriminative power for an early prediction of organ failure and sepsis severity.

Furthermore, APACHE II is frequently used to measure disease severity in intensive care units, but widely adopted APACHE II scoring system has its limitations in predicting the outcome in ARDS. Endocan has high reproducibility of measurement and accessibility of specimens, had a good correlation with APACHE II, and was associated with an increased risk of ARDS death; therefore, it may well complement the APACHE II scoring in outcome prediction and guide therapeutic choices in the early stages of the ARDS aimed at prevention and provide patient benefits using evidence-based therapies.

Plasma endocan Levels elevated dramatically in pneumonia patients with ARDS compared with those without ARDS. It suggested that patients with elevated levels of endocan may develop into ARDS more easily. However, Dr. Mikkelsen et al. found that lower levels of serum endocan on admission are associated with subsequent development of ALI in trauma patients [23]; they explained that it may be associated with endocan-mediated blockade of leukocyte recruitment in the lung. Although trauma and infection may differ clinically and biologically [24], we could prospectively measure the endocan levels of patients with pulmonary infection to identify whether patients with higher levels of endocan are at high risk for ARDS.

The present study has some limitations. First, the number of patients recruited was relatively small, it would be useful to repeat the study on a larger sample of patients in future. Second, the concentration of endocan was measured only initially, in the first 24 h after the inclusion in the study, and the dynamics of concentration during the ARDS evolution has not been evaluated. Third, the study lacks evaluation of the correlation between the plasma endocan and other biomarkers of ARDS. Fourth, we did not evaluate the diagnose performance of endocan associated with ARDS in the at-risk population; therefor, further studies involving large critical ill subjects at risk for ARDS are needed.

In conclusion, our study is one of the few studies to study the predictive value of endocan for ARDS. We have demonstrated that endocan can predict the MODS development and mortality of ARDS independently. It may guide effective rescue therapies such as lung protective ventilation strategy, liquid negative balance management, and organ protective treatment, thus reducing the mortality of ARDS. In addition, combined clinical variables with biological biomarkers such as endocan may play an important role in early therapeutics or preventative approaches for ARDS.

## Figures and Tables

**Table 1:** Baseline demographics, clinical characteristics, and comorbidity of 42 patients with acute respiratory distress syndrome.

	Survivors (*N* = 22)	Nonsurvivors (*N* = 20)	*P* value
Age, years, mean (SD)	63.9 (17.3)	72.5 (10.8)	0.064
Male sex, *n* (%)	13 (59)	14 (70)	0.531
APACHE II score, median (IQR)	21 (18–24)	24 (22–30)	0.03
PaO2/FIO2 ratio, median (IQR)	131 (100–150.5)	89.5 (64–111)	0.000
Etiology of ARDS, *n* (%)			
Pulmonary infection	18 (81.8)	17 (85)	
Bacterial pneumonia	14 (63.6)	15 (75)	
Virus pneumonia	2 (9)	2 (10)	
Active tuberculosis	2 (9)	0 (0)	
Aspiration	0	1 (5)	
Blood transfusion	2 (9)	0 (0)	
Others	2 (9)	2 (10)	
Abdominal infection	1 (4.5)	2 (10)	
Mediastinal abscess	1 (4.5)	0 (0)	
Comorbidity, *n* (%)			
Obstructive airway disease	6 (27.2)	7 (35)	0.741
Hypertension	10 (45.4)	14 (70)	0.131
Cardiovascular disease	8 (36.3)	13 (65)	0.121
Cerebrovascular accident	1 (4.5)	2 (10)	0.598
Diabetes	10 (45.4)	11 (55)	0.758
Duration of Mechanical ventilation, mean (SD)	12 (4.5)	15 (10.5)	0.216
Length of Intensive Care Unit stay, mean (SD)	16 (6.5)	16 (12)	0.846
Length of hospital stay	28.0 (8.0)	17.5 (12.0)	0.002
MODS			
MODS = 2 organs, *n* (%)	6 (27.2)	5 (25)	1.0
MODS = 3 organs, *n* (%)	1 (4.5)	7 (35)	0.018
MODS ≥ 4 organs, *n* (%)	2 (9)	4 (20)	0.4
Shock	3 (13.6)	11 (55)	0.008
Shock (<7 days)	3 (13.6)	7 (35)	0.152
Renal failure	3 (13.6)	10 (50)	0.114
Renal failure (<7 days)	1 (4.5)	5 (25)	0.087
Coagulopathy	2 (9)	7 (35)	0.062
Coagulopathy (<7 days)	0 (0)	6 (30)	0.007
Hepatic dysfunction	6 (27.2)	6 (30)	1.0
Hepatic dysfunction (<7 days)	0 (0)	6 (30)	0.007

APACHE = acute physiology and chronic health evaluation;

ARDS = acute respiratory distress syndrome; MODS = multiple-organ dysfunction syndrome.

*P values for age by the *t*-test and those for APACHE II scores and PaO2/FIO2 by the Mann-Whitney *U* test. Fisher exact tests were applied for categorical variables.

**Table 2:** Comparison of plasma biomarkers between survivors and nonsurvivors of acute respiratory distress syndrome.

	Survivors (*N* = 22)	Nonsurvivors (*N* = 20)	*P* value
Endocan (ng/mL), median (IQR)	3.01 (2.36–4.36)	5.01 (2.98–8.44)	0.017
PCT (ng/mL), median (IQR)	2.59 (0.95–6.25)	9.26 (3.88–16.46)	0.007
CRP (mg/L), median (IQR)	113.4 (85.5–170.3)	139.9 (101.2–196.3)	0.385
WBC (×10^9^/L), median (IQR)	14.58 (8.84–19.50)	15.25 (10.9–19.57)	0.706
N (×10^9^/L), median (IQR)	13.20 (7.70–19.76)	14.08 (9.76–18.80)	0.762

PCT = procalcitonin, CRP = C-reactive protein, WBC = white blood cell, and N = Neutrophil counts.

*P values for these biomarkers by the Mann-Whitney *U* test. *P* value less than 0.05 was considered statistically significant.

**Table 3:** Cox proportional hazards models for mortality prediction by biomarkers and severity scores.

Variable	Univariate Cox model		Multivariate Cox model	
	HR (95% CI)	*P* value	HR (95% CI)	*P* value
Endocan	1.386 (1.171–1.641)	0.000	1.374 (1.150–1.641)	0.000
PaO2/FIO2	0.96 (0.943–0.978)	0.000	0.958 (0.938–0.978)	0.000
CRP	NA	NA	NA	NA
WBC	NA	NA	NA	NA
PCT	1.063 (1.023–1.105)	0.002	NA	NA
APAHCE II	1.155 (1.040–1.282)	0.007	NA	NA

HR = hazard ratio; NA = not applicable; *P* value less than 0.05 was considered statistically significant.

**Table 4:** Areas under receiver operating characteristic curves for plasma biomarker levels in predicting organ dysfunction in patients with acute respiratory distress syndrome.

	Endocan	PCT	CRP	WBC	APACHE II
Organ dysfunction (duration of hospital stay)					
Septic shock	0.772∗	0.624	0.529	0.658	0.770^#^
Renal failure	0.714∗	0.593	0.615	0.432	0.662
Coagulopathy	0.650	0.694	0.444	0.458	0.625
Hepatic dysfunction	0.490	0.535	0.311	0.503	0.575
Organ dysfunction (Within 7 days)					
Septic shock	0.786∗	0.566	0.511	0.531	0.787^#^
Renal failure	0.773∗	0.495	0.620	0.481	0.715
Coagulopathy	0.852^#^	0.718	0.519	0.444	0.715
Hepatic dysfunction	0.690	0.715	0.370	0.519	0.752

*P≦0.05; ^#^ *P*≦0.01.

**Table 5:** Organ dysfunctions in patients with acute respiratory distress syndrome with low or high plasma endocan levels.

	Endocan ≥ 4.96 ng/mL (*n* = 15)	Endocan < 4.96 ng/mL (*n* = 27)	*P* value
Organ dysfunction (duration of hospital stay)				
Septic shock, *n* (%)	9 (60)	5 (18.5)	0.015∗
Renal failure, *n* (%)	8 (53)	5 (18.5)	0.035∗
Coagulopathy, *n* (%)	5 (33)	4 (14.8)	0.242
Hepatic dysfunction, *n* (%)	4 (27)	8 (30)	1.0
Organ dysfunction (Within 7 days)			
Septic shock, *n* (%)	7 (47)	3 (11)	0.020∗
Renal failure, *n* (%)	4 (27)	2 (7.4)	0.164
Coagulopathy, *n* (%)	5 (33)	1 (3.7)	0.016∗
Hepatic dysfunction, *n* (%)	3 (20)	3 (11)	0.649

The occurrence of organ dysfunctions was compared in patient groups with low (<4.96 ng/mL) and high (≥4.96 ng/mL) plasma levels of endocan during hospital stay or within 7 days. Asterisk indicates statistically significant difference between patient groups with low and high plasma endocan levels (*P≦0.05;).

**Table 6:** Demographic and baseline clinical characteristics of pneumonia with ARDS or without ARDS.

	With ARDS (*n* = 35)	Without ARDS (*n* = 44)	*P* value
Age, years, mean (SD)	68 (14)	63.5 (15)	0.194
Male/female	22/15	26/18	0.818
APACHE II score, median (IQR)	23 (19–27)	—	
PaO2/FIO2 ratio, median (IQR)	108 (84–130)	—	
Duration of mechanical ventilation, mean (SD)	13.5 (8)	—	
Length of Intensive Care Unit stay, mean (SD)	15.5 (8.5)	—	
Length of hospital stay, mean (SD)	22 (11)	11 (4)	0.000
Death in hospital, *n* (%)	17 (48.6%)	0	

**Table 7:** Comparison of plasma biomarkers between pneumonia with ARDS and those without ARDS.

	With ARDS (*n* = 35)	Without ARDS (*n* = 44)	*P* value
Endocan (ng/mL), median (IQR)	3.22 (2.47–5.14)	2.45 (2.23–2.79)	0.000
PCT (ng/mL), median (IQR)	4.25 (1.95–9.27)	2.49 (0.55–9.95)	0.186
CRP (mg/L), median (IQR)	135 (88.15–185.08)	124.27 (95.78–165.57)	0.961
WBC (×10^9^/L), median (IQR)	14.78 (11.51–18.22)	12.59 (10.19–16.52)	0.108
N (×10^9^/L), median(IQR)	13.45 (9.65–17.23)	11.07 (8.43–14.52)	0.056

*P values for these biomarkers by the Mann-Whitney *U* test. *P* value less than 0.05 was considered statistically significant.

## References

[1] Phua J, Badia JR, Adhikari NKJ (2009). Has mortality from acute respiratory distress syndrome decreased over time?. *American Journal of Respiratory and Critical Care Medicine*.

[2] Rubenfeld GD, Herridge MS (2007). Epidemiology and outcomes of acute lung injury. *Chest*.

[3] Herridge MS, Tansey CM, Matté A (2011). Functional disability 5 years after acute respiratory distress syndrome. *The New England Journal of Medicine*.

[4] Stüber F, Wrigge H, Schroeder S (2002). Kinetic and reversibility of mechanical ventilation-associated pulmonary and systemic inflammatory response in patients with acute lung injury. *Intensive Care Medicine*.

[5] Ware LB, Koyama T, Billheimer DD (2010). Prognostic and pathogenetic value of combining clinical and biochemical indices in patients with acute lung injury. *Chest*.

[6] Prabhakaran P, Ware LB, White KE, Cross MT, Matthay MA, Olman MA (2003). Elevated levels of plasminogen activator inhibitor-1 in pulmonary edema fluid are associated with mortality in acute lung injury. *The American Journal of Physiology—Lung Cellular and Molecular Physiology*.

[7] Ware LB, Fang X, Matthay MA (2003). Protein C and thrombomodulin in human acute lung injury. *The American Journal of Physiology—Lung Cellular and Molecular Physiology*.

[8] Sato H, Callister MEJ, Mumby S (2004). KL-6 levels are elevated in plasma from patients with acute respiratory distress syndrome. *European Respiratory Journal*.

[9] Determann RM, Royakkers AANM, Haitsma JJ (2010). Plasma levels of surfactant protein D and KL-6 for evaluation of lung injury in critically ill mechanically ventilated patients. *BMC Pulmonary Medicine*.

[10] Nakamura T, Sato E, Fujiwara N, Kawagoe Y, Maeda S, Yamagishi S (2011). Increased levels of soluble receptor for advanced glycation end products (sRAGE) and high mobility group box 1 (HMGB1) are associated with death in patients with acute respiratory distress syndrome. *Clinical Biochemistry*.

[11] Agrawal A, Matthay MA, Kangelaris KN (2013). Plasma angiopoietin-2 predicts the onset of acute lung injury in critically ill patients. *American Journal of Respiratory and Critical Care Medicine*.

[12] Uchida T, Shirasawa M, Ware LB (2006). Receptor for advanced glycation end-products is a marker of type I cell injury in acute lung injury. *The American Journal of Respiratory and Critical Care Medicine*.

[13] Calfee CS, Eisner MD, Parsons PE (2009). Soluble intercellular adhesion molecule-1 and clinical outcomes in patients with acute lung injury. *Intensive Care Medicine*.

[14] Lassalle P, Molet S, Janin A (1996). ESM-1 is a novel human endothelial cell-specific molecule expressed in lung and regulated by cytokines. *Journal of Biological Chemistry*.

[15] Bechard D, Meignin V, Scherpereel A (2000). Characterization of the secreted form of endothelial-cell-specific molecule 1 by specific monoclonal antibodies. *Journal of Vascular Research*.

[16] Béchard D, Scherpereel A, Hammad H (2001). Human endothelial-cell specific molecule-1 binds directly to the integrin CD11a/CD18 (LFA-1) and blocks binding to intercellular adhesion molecule-1. *Journal of Immunology*.

[17] Scherpereel A, Depontieu F, Grigoriu B (2006). Endocan, a new endothelial marker in human sepsis. *Critical Care Medicine*.

[18] Mihajlovic DM, Lendak DF, Brkic SV (2014). Endocan is useful biomarker of survival and severity in sepsis. *Microvascular Research*.

[19] Ranieri VM, Rubenfeld GD, Thompson BT (2012). Acute respiratory distress syndrome: the Berlin definition. *The Journal of the American Medical Association*.

[20] Knaus WA, Draper EA, Wagner DP, Zimmerman JE (1985). APACHE II: a severity of disease classification system. *Critical Care Medicine*.

[21] Terpstra ML, Aman J, van Nieuw Amerongen GP, Groeneveld AB (2014). Plasma biomarkers for acute respiratory distress syndrome: a systematic review and meta-analysis. *Critical Care Medicine*.

[22] Lee H (2013). Procalcitonin as a biomarker of infectious diseases. *Korean Journal of Internal Medicine*.

[23] Mikkelsen ME, Shah CV, Scherpereel A (2012). Lower serum endocan levels are associated with the development of acute lung injury after major trauma. *Journal of Critical Care*.

[24] Calfee CS, Eisner MD, Ware LB (2007). Trauma-associated lung injury differs clinically and biologically from acute lung injury due to other clinical disorders. *Critical Care Medicine*.

